# Adsorption Behavior of High Stable Zr-Based MOFs for the Removal of Acid Organic Dye from Water

**DOI:** 10.3390/ma10020205

**Published:** 2017-02-20

**Authors:** Ke-Deng Zhang, Fang-Chang Tsai, Ning Ma, Yue Xia, Huan-Li Liu, Xue-Qing Zhan, Xiao-Yan Yu, Xiang-Zhe Zeng, Tao Jiang, Dean Shi, Chang-Jung Chang

**Affiliations:** 1 Hubei Key Laboratory of Polymer Materials, Key Laboratory for the Green Preparation and Application of Functional Materials (Ministry of Education), Hubei Collaborative Innovation Center for Advanced Organic Chemical Materials, School of Materials Science and Engineering, Hubei University, Wuhan 430062, China; zkd19910705@hotmail.com (K.-D.Z.); maning@whu.edu.cn (N.M.); xia.yue1990@hotmail.com (Y.X.); pangchoudan521@163.com (H.-L.L.); m18272163856@163.com (X.-Q.Z.); m13477071614@163.com (X.-Y.Y.); xiangzhe1103@163.com (X.-Z.Z.); jiangtao@hubu.edu.cn (T.J.); deanshi2012@yahoo.com (D.S.); 2 Material and Chemical Research Laboratories, Industrial Technology Research Institute, Hsinchu 31040, Taiwan; changcj@itri.org.tw

**Keywords:** UiO-66, adsorption behavior, adsorption mechanism, acid orange 7

## Abstract

Zirconium based metal organic frameworks (Zr-MOFs) have become popular in engineering studies due to their high mechanical stability, thermostability and chemical stability. In our work, by using a theoretical kinetic adsorption isotherm, we can exert MOFs to an acid dye adsorption process, experimentally exploring the adsorption of MOFs, their external behavior and internal mechanism. The results indicate their spontaneous and endothermic nature, and the maximum adsorption capacity of this material for acid orange 7 (AO7) could be up to 358 mg·g^−1^ at 318 K, estimated by the Langmuir isotherm model. This is ascribed to the presence of an open active metal site that significantly intensified the adsorption, by majorly increasing the interaction strength with the adsorbates. Additionally, the enhanced π delocalization and suitable pore size of UiO-66 gave rise to the highest host–guest interaction, which further improves both the adsorption capacity and separation selectivity at low concentrations. Furthermore, the stability of UiO-66 was actually verified for the first time, through comparing the structure of the samples before and after adsorption mainly by Powder X-ray diffraction and thermal gravimetric analysis.

## 1. Introduction

Water is one of the most important natural resources for human beings to live and develop; it is particularity indispensable and irreplaceable. However, with the development of global industrialization since the industrial revolution, the quantity and quality of our water resources is deteriorating continuously, and various contaminants, such as toxic organic compounds, fluoride, heavy metal ions, arsenic compound, and so on, have been detected at the maximum contaminant level in waste water [1,2]. Therefore, the issue of effectively solving the water problem has attracted much attention in our society; a society which will face great challenge in the upcoming decades [3,4]. Several water treatment options, such as biological, physical and chemical methods, have been employed successfully to solve or alleviate the contaminants problem [5,6,7,8,9,10,11]. Among these methods, adsorption over porous materials is generally considered to be one of the most promising approaches for water purification over the past decades as it is efficient, environmentally friendly and low-cost [12].

The traditional porous adsorbents, including zeolites [13], activated carbon [14], natural clays [15], polymer-based porous materials [16], and so on [17,18] often used to handle water pollution. However, these traditional porous materials almost always have shortcomings, including low capacity, weak interaction and difficulty in regeneration in practical applications [19,20,21]. So, the new porous materials with high capacity, selectivity, lower cost and easier regeneration are still desirable. In order to overcome these obstacles, on the one hand, these traditional porous materials can be modified to improve their performance; [22,23,24] on the other hand, new materials have been prepared to replace the conventional porous materials [25,26,27,28]. Metal-organic frameworks (MOFs) mainly result from the inorganic metal ions or metal clusters coordinated with bidentate or multidentate aromatic organic ligands to form three-dimensional network crystal structures. As a class of advanced crystalline porous materials, MOFs have been considered to be the most promising candidates to replace the conventional porous materials in pollutant removal due to their diverse structure and compositions, high surface area, tunable pore size, numerous active metal sites and so on [29,30,31,32]. In recent years, the study on MOFs for wastewater treatment has mainly been focused on their water stability, regeneration, and the effects of MOFs’ structure, such as pore size, functional group and active metal sites, on the adsorption performance [25,33,34]. However, investigation of the adsorption behavior and affinity of MOFs for contaminants is also necessary for developing new adsorbents and realizing the practical application.

In numerous MOFs, UiO-66—as a remarkable hydro-stability material—is comprised of inorganic nodes Zr_6_O_4_(OH)_4_ coordinated with terephthalate ligands to form a porous cubic framework [35]. This three-dimensional (3D) porous solid contains two types of cage, in which each centric octahedral cage is surrounded by eight corner tetrahedral cages (free diameters of approximate 11 and 8 Å for the two types of cages, respectively) connected through narrow windows (approximate 6 Å) (Figure S1, ESI) [36]. Furthermore, it is reported that the size effect of UiO-66 powder crystals is from hundreds of nanometers to dozens of nanometers [37]. It is worth mentioning that zirconium has a high affinity towards oxygen ligands and Lewis acid character, making these bridges very strong, resulting in UiO-66 having high thermal stability and its structure remaining unaltered in numerous solvents such as water, dimethyl formamide (DMF), benzene and acetone, compared to other MOF structures. This stability is also the basic factor for MOFs to be applied to water purification. Moreover, the high-valence zirconium cations, as open metal sites contained in a secondary building unit, can target specific adsorption behavior.

**Figure 1:**
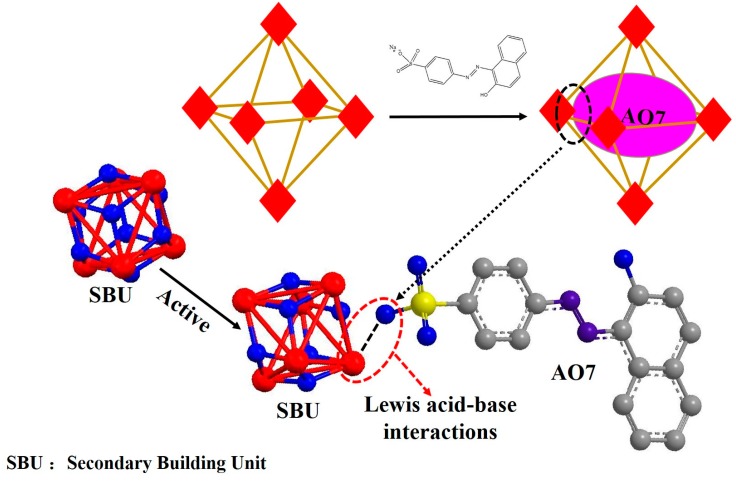
Illustration of the potential mechanism of acid orang 7 (AO7) adsorbed into UiO-66.

On the basis of the performance of UiO-66, we turned our attention to the adsorption behavior and adsorption mechanism, especially the affinity of UiO-66 for specific pollutants depended on Lewis acid-base character. In this work, UiO-66 was employed to study the adsorption progress and the interaction by using a kind of acid dye, acid orang 7 (AO7), whose three dimensions are 5.44, 10.03 and 15.67 Å respectively [38], exploring the external behavior and internal mechanism between UiO-66 and dyes. The results show that the adsorption is a spontaneous process of thermodynamics, and obeys the pseudo-second order kinetic model. The adsorption isotherm study reveals that the adsorption is well fitted by the Langmuir isotherm model with monolayer adsorption, and the maximum adsorption capacity of this MOF material for AO7 is estimated to be up to 358 mg·g^−1^ at 318 K. Finally, the Lewis acid-base interaction between AO7 and UiO-66 is verified and can be described as shown in Figure 1, in which the zirconium ions, as an open active site, can coordinate with the sulfosalt contained in AO7. The strength of the Lewis acid-base interaction of Zr-(-SO_3_^−^) is higher than Zr-(H_2_O)/Zr-(DMF) but less than Zr-(-CO_2_^−^). Therefore, UiO-66 have complete crystal structure during the adsorption process. The zirconium ion with Lewis acid character in UiO-66 is encompassed by water molecules, because a lot of water molecules compete with the AO7 molecule to impede the formation of a complex between UiO-66 and AO7 during the initial period. Over time, the AO7 molecule spreads to the surface of UiO-66, and replaces the water molecule to form a relatively stable complex compound.

## 2. Results and Discussion

### 2.1. Powder X-ray Diffraction (PXRD)

**Figure 2:**
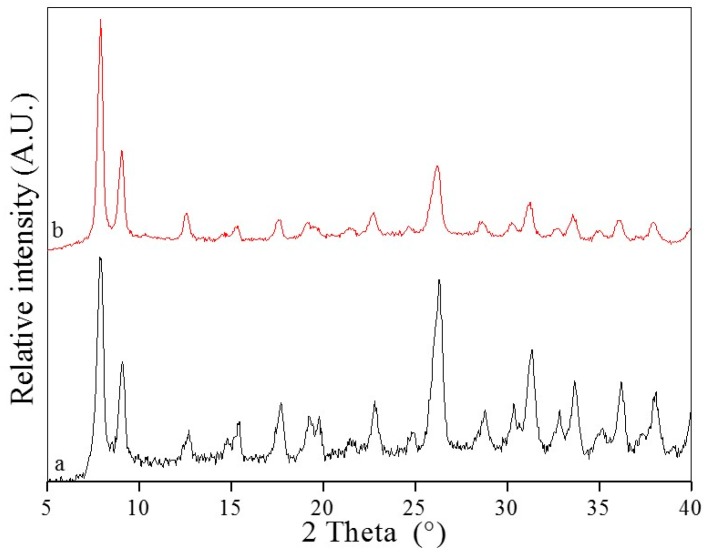
The Powder X-ray diffraction (PXRD) of UiO-66 (**a**) and UiO-66-AO7 (**b**).

Since stability is the basic factor for MOFs to be applied to gas storage, catalysis, water treatment, drug delivery, fluorescence sensing and so on, the samples of UiO-66 before and after adsorption were collected and contrastively analyzed using Powder X-ray diffraction (PXRD). The sample of AO7 adsorbed into UiO-66 is denoted as UiO-66-AO7. The PXRD pattern of UiO-66 and UiO-66-AO7 are shown in Figure 2. In curve a of Figure 2, the well-defined diffraction peaks of the as-synthesized powder UiO-66 can be observed, and they are in good agreement with the reported literature, indicating the symmetric cubic structure and high crystallinity [39]. To examine the framework structure stability after adsorption of AO7 into UiO-66, PXRD data of UiO-66-AO7 were also collected. Compared with the UiO-66, there is no significant change in peak positions for the UiO-66-AO7, revealing the stability of the structure of UiO-66 after adsorption of AO7. The stability of UiO-66 can also be testified from the characterization of Fourier transform infrared spectroscopy (FT-IR) and Thermal gravitational analysis (TGA). After adsorption, the characterization peaks of UiO-66 still exist (Figure S2, ESI), and the decomposition temperature of UiO-66-AO7 is basically consistent with UiO-66 in each stage (Figure S3, ESI).

### 2.2. Adsorption Kinetic Studies

In the first, the maximum absorption wavelength of AO7 was obtained (Figure S4, ESI), and the standard curve with absorbance versus concentration was depicted (Figure S5, ESI). And then, the adsorption kinetic experiments were executed at 298 K, in which a 5 mg sample of powdered UiO-66 and 50 mL of AO7 aqueous solution were placed in a 100 mL beaker.

The amount of dye adsorbed into adsorbents (*q_t_*, mg·g^−1^) was determined by the following Equation (1) [40].

(1) $$q_{t}=\frac{\left(C_{0}-C_{t}\right)v_{0}}{m}$$

where *C*_0_ is the initial concentration of dye in solution (mg·L^−1^); *C_t_* is the concentration of dyes at time t in solution (mg·L^−1^). The *v*_0_ is the volume of dye solution (L); *m* is the mass of the adsorbent (g).

**Figure 3:**
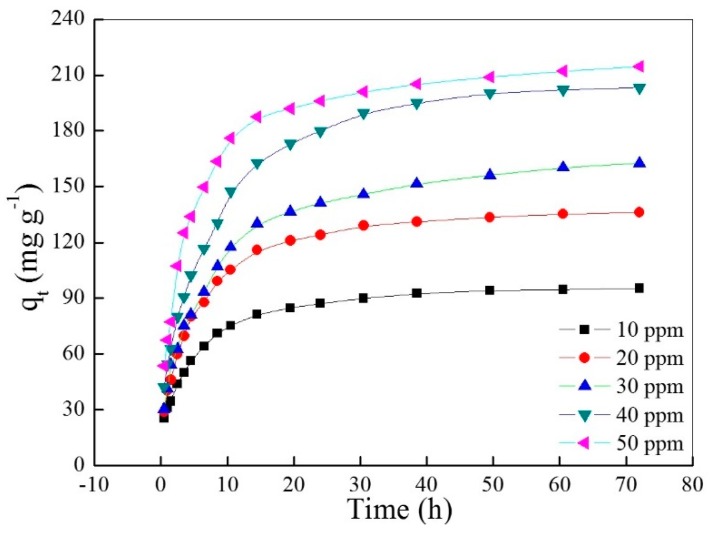
Effects of contact time and initial concentration of AO7 aqueous solution on the adsorptive capacity.

The curve of adsorptive capacity versus time is shown in Figure 3. Obviously, the adsorptive capacity increases as the contact time or initial concentration increase, and the adsorption rate decreases as the contact time increased and slowly reached zero at last. Moreover, the adsorption rate increases as the initial dye concentration increases in the initial period, in which the diffusion of the dye molecule as a rate-controlling step plays a dominant role.

In order to understand the potential reaction mechanism, the changes of adsorptive capacity with time were simulated by the theoretical kinetic models. In this study, a pseudo-first order model and a pseudo-second order kinetic model were employed to be tested [41,42].

The pseudo-first order and pseudo-second order kinetic models were described by the following linear Equations (2) and (3), respectively:

(2) $$\ln\left(q_{e}-q_{t}\right)=\ln q_{e}-k_{1}t$$

(3) $$\frac{t}{q_{t}}=\frac{1}{k_{2}q_{e}^{2}}+\frac{t}{q_{e}}$$

where *t* is the contact time (h); *k*_1_ and *k*_2_ are the rate constant of the pseudo-first order and pseudo-second order kinetic models, respectively.

**Figure 4:**
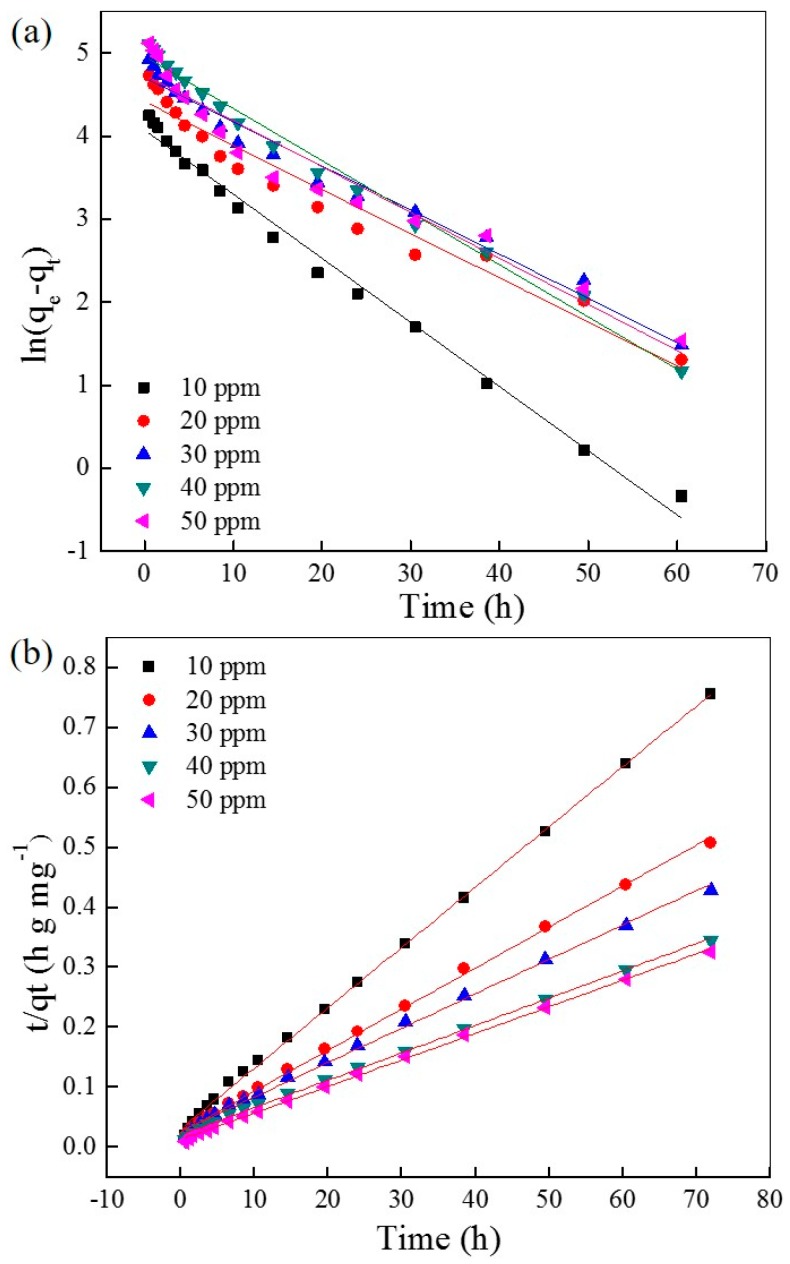
Simulation of the pseudo-first order (**a**) and pseudo-second order (**b**) kinetic models of AO7 adsorbed into UiO-66.

The fitting plots with the pseudo-first order and pseudo-second order kinetic models are shown in Figure 4a,b, respectively, and the relative parameters are listed in Table 1 and Table 2, respectively. The correlation coefficients (*R*^2^) of the pseudo-second order kinetic model are higher than the pseudo-first order kinetic model under varying initial AO7 concentrations, and are very close to 1 (*R*^2^ > 0.999). This result suggests that the adsorption progress of AO7 into UiO-66 is more suitable for the pseudo-second order kinetic model, indicating a chemisorption. This result also suggests that the strong ion interaction between the zirconium ion and sulfonate ion is very possible, which plays a dominant role over a long period as a rate-controlling step.

### 2.3. Adsorption Isotherm Studies

**Figure 5:**
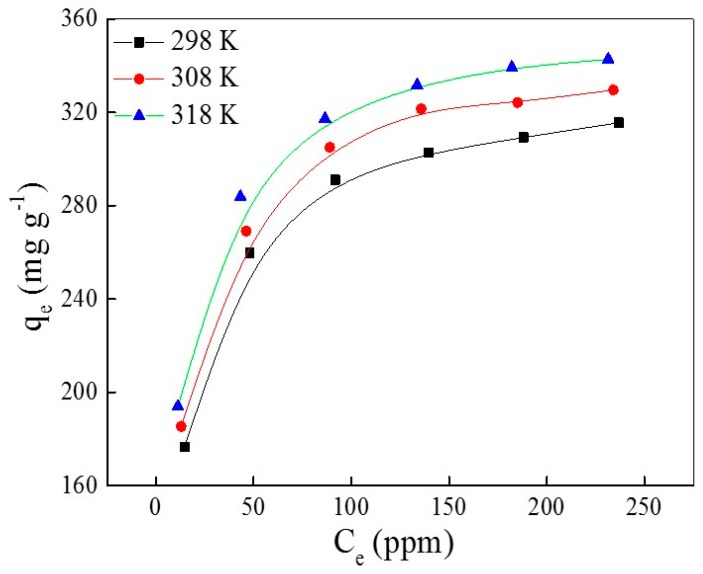
The plots of *q_e_* versus *C_e_* at different temperatures.

The adsorption isotherm is one of most important data to measure the saturated adsorption capacity of any adsorbent, explore the adsorption rationale and the practical application of adsorbents. An adsorption isothermal experiment of AO7 adsorbed into UiO-66 is conducted at varying initial AO7 concentrations (from 50 to 300 ppm) over 72 h under different temperatures. The plots with adsorption capacity versus concentration at equilibration under different temperatures are shown in Figure 5. Obviously, the adsorption capacity at equilibration increases with the increasing of temperature, owing to the endothermic process that dissociates the water molecule from the surface of AO7 or secondary building units (SBU) of UiO-66.

In order to thoroughly understand the adsorption behavior, the experimental dates were evaluated by two generally used isothermal models—the Langmuir and Freundlich models—in this study. The Langmuir isothermal model is based on the assumption of monolayer adsorption, in which the adsorbate only combines with a finite number of open active sites that are identical and equivalent [43]. However, the Freundlich isothermal model is only an empirical model whose earliest known relationship describes the non-ideal and reversible adsorption, which can be applied to multilayer adsorption without being restricted to the formation of a monolayer [44]. The Langmuir and Freundlich isothermal models were described by the following linear Equations (4) and (5), respectively:

(4) $$\frac{C_{e}}{q_{e}}=\frac{C_{e}}{q_{s}}+\frac{1}{q_{s}K_{L}}$$

(5) $$\ln q_{e}=\ln K_{F}+\frac{1}{n_{F}}\ln c_{e}$$

where *q_s_* (mg·g^−1^) is the amount of adsorption of AO7 at a theoretical saturation capacity; *K_L_* and *K_F_* are the constants of the Langmuir and Freundlich isotherm model, respectively; *n_F_* is the intensity of the adsorption.

**Figure 6:**
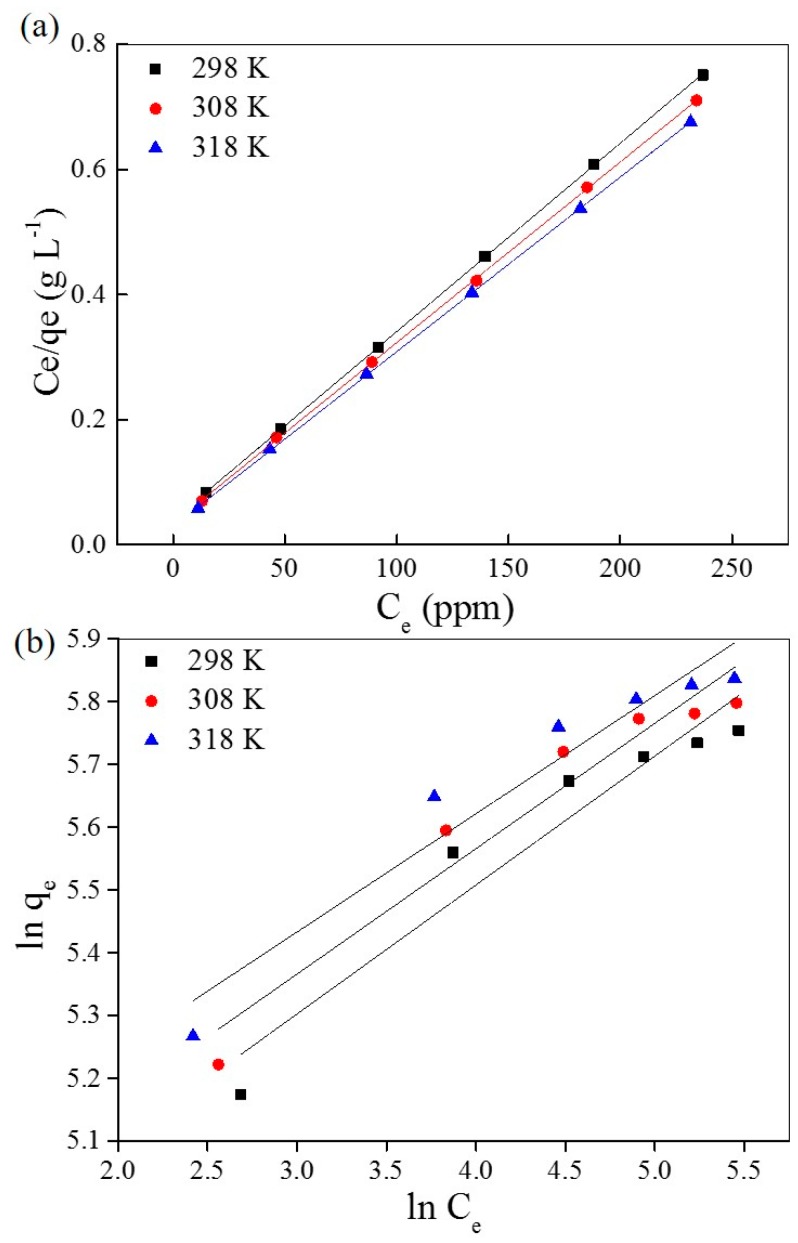
Simulation of the Langmuir (**a**) and Freundlich (**b**) isothermal models of AO7 adsorbed into UiO-66.

The simulation curves with the Langmuir and Freundlich isothermal models are shown in Figure 6a,b, respectively, and the relative permeants are listed in Table 3 and Table 4, respectively. The correlation coefficients (*R*^2^) of the Langmuir isotherm model are higher than the Freundlich isothermal model—very close to 1 (*R*^2^ > 0.9999) under different temperatures. This provides strong evidence that the adsorption of AO7 into UiO-66 follows the Langmuir isothermal model, which indicates that the adsorption takes place on homogeneous sites that are identical and energetically equivalent. However, the slight variation of K_L_ with the temperature changes indicates that the affinity of binding sites is independent of temperature; this is mainly to overcome the barrier of water molecules. Furthermore, the maximum adsorption capacity of UiO-66 for AO7 is estimated to be up to 358 mg·g^−1^ at 318 K.

### 2.4. Adsorption Thermodynamics

From the adsorption isotherm, the adsorption capacity of AO7 into UiO-66 increases as the temperature increases, revealing an endothermic process. The thermodynamics parameters such as Gibbs free energy change (Δ*G*^0^), enthalpy (Δ*H*^0^) and entropy (Δ*S*^0^) can be calculated from the adsorption isotherms parameters and further reveal the adsorption mechanism. The thermodynamics parameters were determined using the following equations [45]:

(6) $$△G^{0}=-RTLnK_{L}$$

(7) $$△G^{0}=△H^{0}-TS^{0}$$

**Figure 7:**
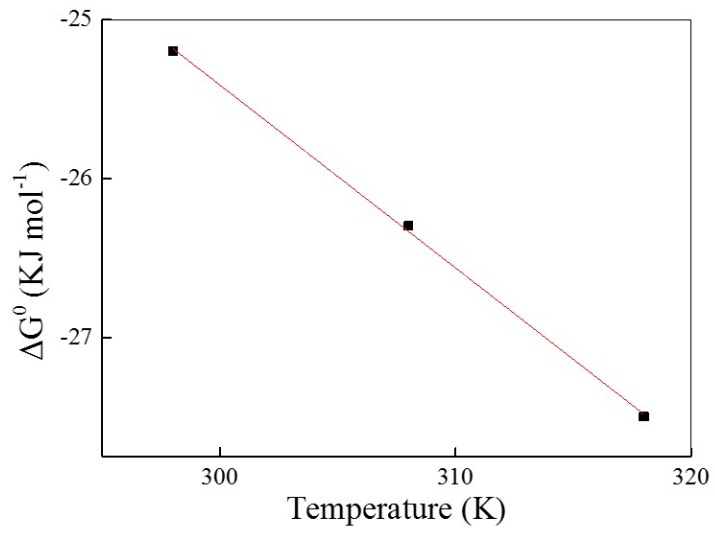
The plot of Δ*G*^0^ versus temperature.

The Gibbs free energy change (Δ*G*^0^) of the adsorption of AO7 into UiO-66 obtained at all the temperatures was listed in Table 4. As shown in Figure 7, Δ*H*^0^ and Δ*S*^0^ were determined from the slope and intercept of the Δ*G*^0^ versus temperature plot and are also tabulated in Table 4. Obviously, the negative values of Δ*G*^0^ at all temperatures and the positive values of Δ*H*^0^ (9.09 kJ·mol^−1^) indicate the spontaneous and endothermic nature of AO7 adsorbed into UiO-66 [46]. Furthermore, the positive value of Δ*S*^0^ (115 J·mol^−1^·K^−1^) reflects the affinity of UiO-66 for AO7 as well as an increased randomness at the solid-solution interface during adsorption.

## 3. Conclusions

In summary, UiO-66 was successfully synthesized by adding concentrated hydrochloric acid as accelerant, promoting crystal growth under a facile condition with low temperature (<353 K). The Lewis acid character of zirconium ions in the SBU of UiO-66, as open active sites, was studied for the removal of AO7 from aqueous solution. The results show that the adsorption progress obeys the pseudo-second order kinetic model, and can be described as the Langmuir isotherm. Moreover, the maximum adsorption capacity of this new MOF material was calculated to be 358 mg·g^−1^ at 318 K by the Langmuir isotherm model. The negative values of Δ*G*^0^ at all the temperatures and the positive values of Δ*H*^0^ (9.09 kJ·mol^−1^) indicates the spontaneous and endothermic nature of AO7 adsorbed into UiO-66. On the other hand, interestingly, the size of the target molecule (acid orange 7) is smaller than the window size of UiO-66, due to the possibility of pores inside and on the surface of MOF crystals. Based on these premises, it is reasonable to believe that the target molecule on the surface of MOF crystals will diffuse into the pores over an adsorption equilibrium point. In addition, the stability of UiO-66 and the moderate Lewis acid-base interaction makes it possible for UiO-66 to replace traditional porous materials applied in water treatment.

## Figures and Tables

**Table 1:** Pseudo-first order kinetic parameters for AO7 adsorption into UiO-66.

*C*_0_ ppm	*q_e_* mg·g^−1^	*k*_1_ 10^−2^·h^−1^	*R*^2^
10	58.6	7.72	0.9899
20	82.6	5.31	0.9510
30	110	5.31	0.9755
40	142	6.27	0.9878
50	114	5.53	0.9363

**Table 2:** Pseudo-second order kinetic parameters for AO7 adsorption into UiO-66.

*C*_0_ ppm	*q_e_* mg·g^−1^	*k*_2_ 10^−3^ g·mg^−1^·h^−1^	*R*^2^
10	99.5	3.11	0.9985
20	146	1.82	0.9973
30	175	1.21	0.9979
40	219	0.987	0.9991
50	226	1.50	0.9990

**Table 3:** Parameters of the Langmuir isotherm for AO7 adsorbed into UiO-66 at different temperatures.

Temperature (K)	*q_s_* (mg·g^−1^)	*K_L_* (10^4^ L·mol^−1^)	*R*^2^	Δ*G*^0^ (kJ·mol^−1^)	Δ*H*^0^ (kJ·mol^−1^)	Δ*S*^0^ (J·mol^−1^·K^−1^)
298	332	2.64	0.9999	−25.2	9.09	115
308	346	2.93	0.9999	−26.3		
318	358	3.32	0.9999	−27.5		

**Table 4:** Parameters of the Freundlich isotherm for AO7 adsorbed into UiO-66 at different temperatures.

Temprature (K)	*K_F_* (mg·g^−1^) (L·mg^−1^)^n^	*n_F_*	*R*^2^
298	108	4.87	0.9092
308	118	5.01	0.9225
318	130	5.31	0.9229

## Supplementary Materials

The following are available online at www.mdpi.com/1996-1944/10/2/205/s1. Figure S1: Schematic illustration of the UiO-66(Zr) structure (a). UiO-66(Zr) contains two cage types: tetrahedral cages (b) and octahedral cages (c). Structure (d) shows the secondary building units. Zirconium, oxygen, carbon and hydrogen atoms are red, blue, gray, and white, respectively, Figure S2: The infrared spectra of UiO-66 (a) and UiO-66-AO7 (b), Figure S3: Thermal properties of UiO-66 (a) and UiO-66-AO7 (b), Figure S4: UV-vis spectrum of AO7 aqueous solution, Figure S5: The standard curve of AO7 aqueous solution.
