# Supplementary material for: Adsorption Behavior of High Stable Zr-Based MOFs for the Removal of Acid Organic Dye from Water
Source: Materials (Basel). 2017 Feb 20;10(2):205. doi: 10.3390/ma10020205 (PMC5459129; doi:10.3390/ma10020205)
Supplement: Supplementary file 1 [file materials-10-00205-s001.pdf]

Supplementary Materials: Adsorption Behavior of High Stable Zr-Based MOFs for the Removal of Acid Organic Dye from Water

Ke-Deng Zhang, Fang-Chang Tsai, Ning Ma, Yue Xia, Huan-Li Liu, Xue-Qing Zhan, Xiao-Yan Yu, Xiang-Zhe Zeng, Tao Jiang, Dean Shi and Chang-Jung Chang

The Structure of UiO-66(Zr)

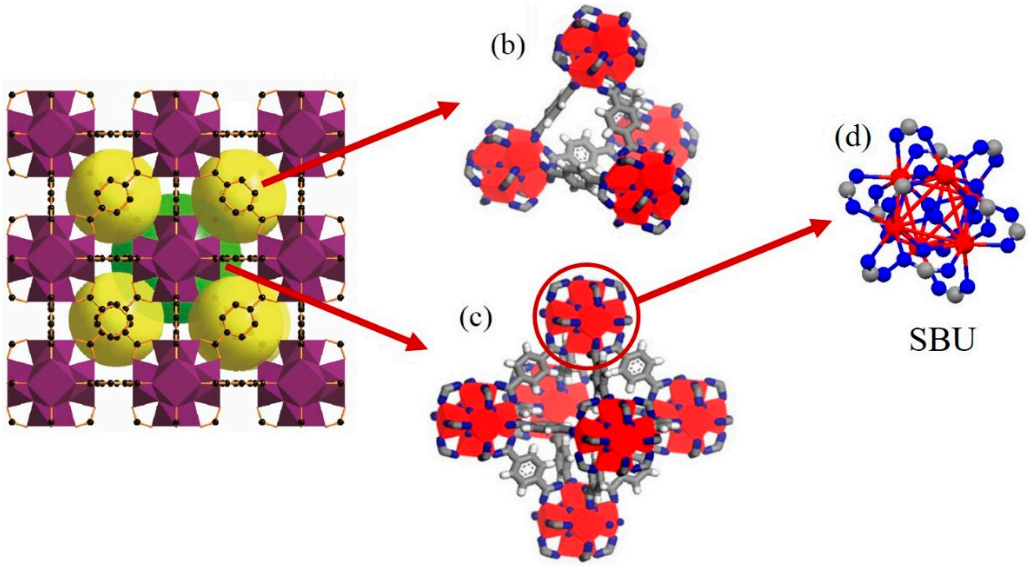

Figure S1. Schematic illustration of the UiO-66(Zr) structure (**a**). UiO-66(Zr) contains two cage types: tetrahedral cages (**b**) and octahedral cages (**c**). Structure (**d**) shows the secondary building units. Zirconium, oxygen, carbon and hydrogen atoms are red, blue, gray, and white, respectively.

General Procedures, Materials, and Instrumentations

Table S1. Reagent specifications and producing area.

Reagent	Purity	Manufacturer	Producing Area
Terephthalic acid (H_2BDC)	99%	Sinopharm	Shanghai, China
Zirconium (IV) chloride ($ZrCl_4$)	≥99.9%	Aladdin	Shanghai, China
N,N′-dimethylformamide (DMF)	≥99.5%	Sinopharm	Shanghai, China
Hydrochloric acid (HCl)	36%–38%	Sinopharm	Shanghai, China
Anhydrous ethanol (EtOH)	≥99.7%	Sinopharm	Shanghai, China

All the reagents involved in this study were commercially available and directly used without any purification.

Powder X-ray diffraction (PXRD) patterns were obtained using a Bruker D8 focus diffractometer (Bruker, Germany). Measurements were made over a range of 2° < 2θ < 40° in 0.05 step size at a scanning rate of 1 deg/min using Cu Kα radiation.

Chemical structure analysis was performed by Nicolet iS50 Fourier transform infrared spectra (FT-IR, ThermoFisher, Waltham, MA, USA). Sixteen scans were coadded in order to achieve an acceptable signal-to-noise ratio. In all cases, the spectral resolution was maintained at 4 cm^{-1}. Measurements were made over a range of 4000–500 cm^{-1} at room temperature in KBr pellets.

The thermal gravitational analysis (TGA) of UiO-66 and UiO-66-AO7 was performed on a TGA 1 Star^e System (Mettler Toledo, Switzerland) interfaced with a PC using Star^e software (version 9.10).

Measurements were made over a range of 30–800 °C and heated at a rate of 10 °C·min^{-1} under a nitrogen atmosphere.

The absorbance measurements of all AO7 aqueous solutions were performed on a UV-visible spectrophotometer (TU-1810DSPC, Beijing Purkinje General Instrument Co., Ltd., Beijing, China) with a PC, using software (UVWin 6).

Preparation of UiO-66 and AO7 Aqueous Solution

A specimen of nanosized UiO-66 was synthesized according to the reported procedures.[1] Briefly, $ZrCl_4$ and H_2BDC were dissolved in the mixture solvent ($v_{(DMF)}$:$v_{(HCl)}$ = 5:1) and dimethyl formamide (DMF), respectively; the molar ratio of $ZrCl_4$ to BDC was 1:1.4. Afterwards, two solutions were mixed and heated at 80 °C for 24 h. After cooling to ambient temperature, the precipitate was assembled by filtration under reduced pressure and then washed with DMF and EtOH three times. Finally, the specimen was dried under vacuum overnight at 90 °C, then heated up to 150 °C; the sample was kept at this temperature for 3 h. To reduce thermal motion and obtain a more accurate structure, the sample was then cooled to 25 °C and the crystal was exposed to the atmosphere, which produces a coordinatively unsaturated site at the zirconium center.

Varying concentrations of AO7 standard solution were prepared. Specific processes are as follows: firstly, 1.00 g AO7 was dissolved in a 1000 ml volumetric flask using ultrapure water to give 1000 ppm AO7 standard solution; secondly, AO7 standard solution (1000 ppm) was diluted serially using ultrapure water to give different concentrations of AO7 standard solution including 10, 20, 30, 40, 50, 100, 200, 300, 400 and 500 ppm.

FT-IR Spectra of UiO-66 as-Synthesized and UiO-66-AO7

Figure S2 summarizes the effect on the vibrational properties (IR) of UiO-66 after adsorption. In curve a of Figure S2, the intense and broad peak at 3360 cm^{-1} is owing to the adsorbed water condensed inside the cavities of UiO-66. The weak peaks at 3050 cm^{-1}–2850 cm^{-1} are assigned to the symmetrical and asymmetrical stretching vibration of the C–H bond in the benzene ring and DMF, while the intense doublet at 1581 cm^{-1} and 1398 cm^{-1} is respectively assigned to the in- and out-of-phase stretching modes of the carboxylate group (C=O). Moreover, the characteristic peak of Zr-O is visible at 750 cm^{-1}–500 cm^{-1} [2]. Comparing the two curves, the characteristic peak of DMF at 1657 cm^{-1} disappears in the infrared spectra of UiO-66-AO7, illustrating that the DMF is replaced by the AO7 coordinating in the UiO-66 after adsorption. In addition to this, in the infrared spectrum of UiO-66-AO7 there is no significant change, indicating the stability of the structure of UiO-66.

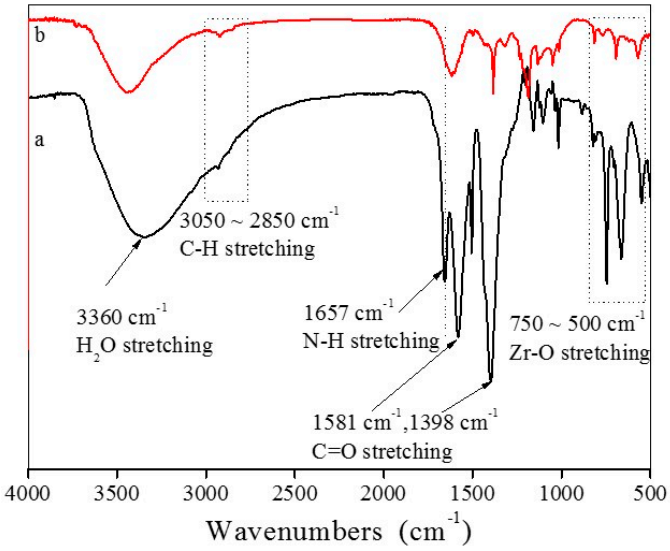

Figure S2. The infrared spectra of UiO-66 (**a**) and UiO-66-AO7 (**b**).

Thermal Gravitational Analysis of UiO-66 and UiO-66-AO7

To investigate the thermal stability of UiO-66 after the adsorption for AO7, their TGA plots were measured in the nitrogen condition and compared for the decomposition temperature in each stage, as shown in Figure S3. Obviously, the decomposition temperature of UiO-66-AO7 is basically consistent with UiO-66 in each stage, indicating that the AO7 is adsorbed into the cavities of UiO-66. In the TGA plot of UiO-66, the thermal gravimetric of UiO-66 can be divided into three stages [1]. The first step, the weight loss is about 9 wt. % from 30 to 100 °C, attributed to the initial solvent loss; the second step, the weight loss is also about 9 wt. % from 200 to 350 °C, attributed to the dehydration of the $Zr_6O_4(OH)_4$ nodes to Zr_6O_6. The last step, the weight loss is about 42 wt. % from 400 to 600 °C, attributed to the decomposition of the organic linkers. For the thermal gravimetric of UiO-66-AO7, the thermal decomposition of AO7 is mainly in the last step, because the adsorbed AO7 is condensed inside the cavities of UiO-66 [3].

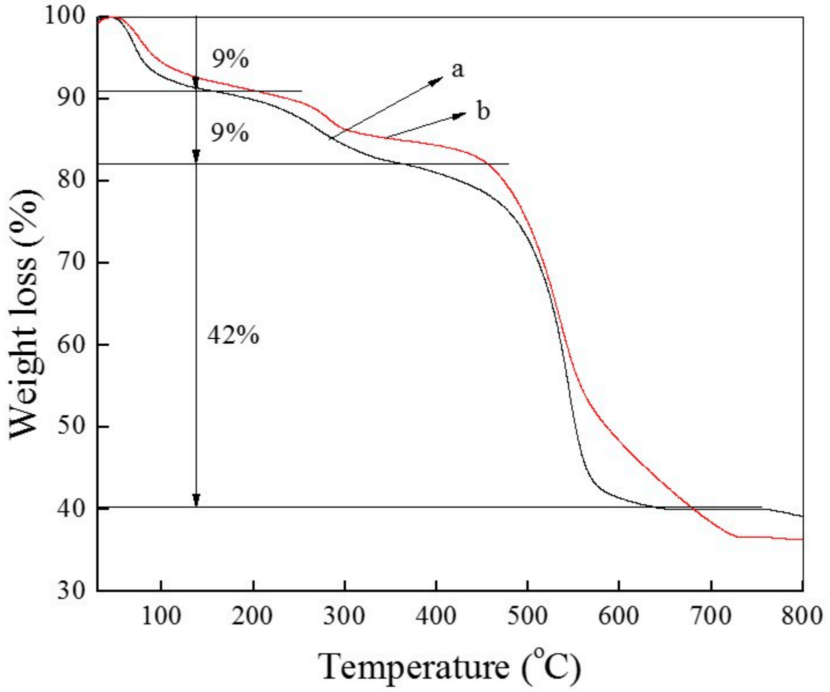

Figure S3. Thermal properties of UiO-66 (**a**) and UiO-66-AO7 (**b**).

The UV-vis Spectrum and the Standard Curve of AO7 Aqueous Solution

The maximum absorption wavelength was obtained through UV-visible full wavelength scanning to monitor the adsorption process. As shown in Figure S4, the characteristic adsorption of AO7 solution was about at 484.5 nm. The standard curve with absorbance versus concentration was depicted via measuring the absorbance of different concentrations of AO7 aqueous solutions. As shown in Figure S5, the absorbance is linearly relative to the concentration of AO7 aqueous solution, no more 40 ppm, and the correlation coefficient is 0.9998, which obeys Lambert–Beer Law.

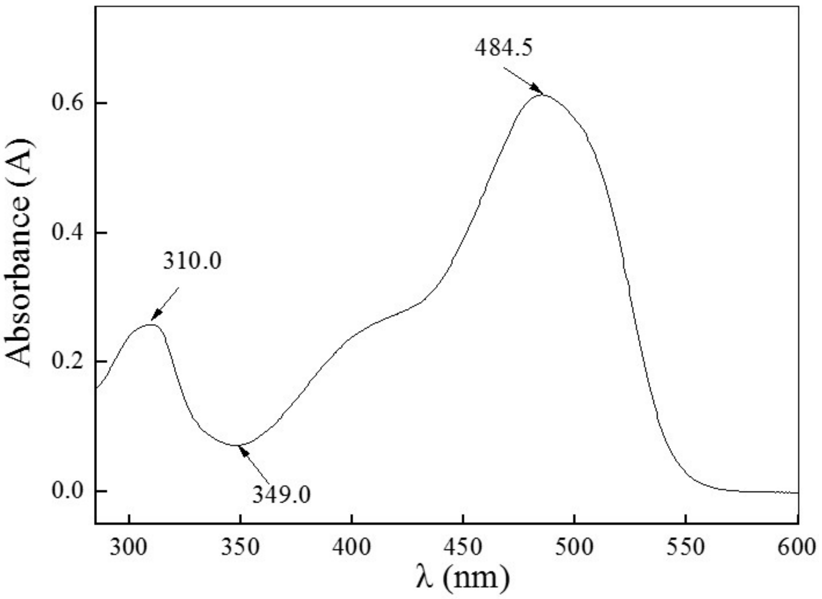

Figure S4. UV-vis spectrum of AO7 aqueous solution.

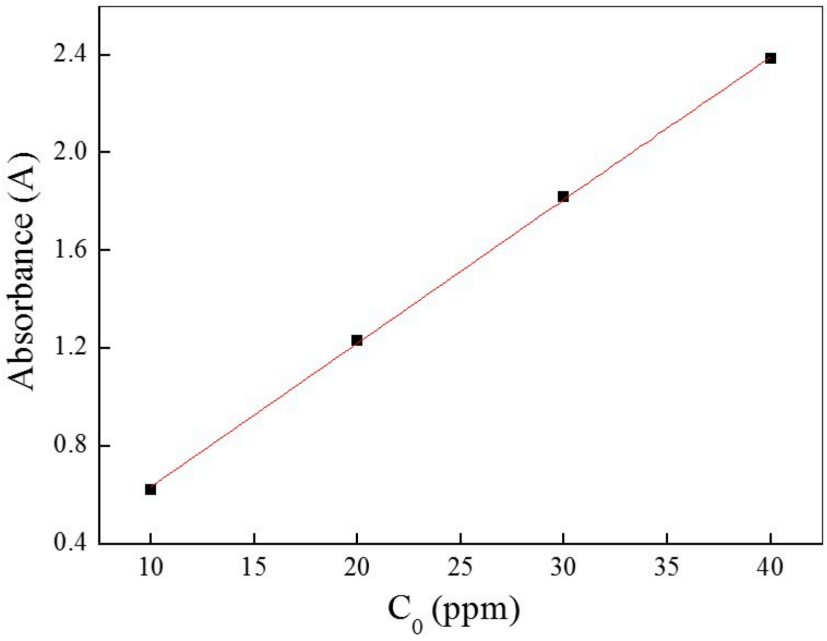

Figure S5. The standard curve of AO7 aqueous solution.

References

1. Katz, M.J.; Brown, Z.J.; Colón, Y.J.; Siu, P.W.; Scheidt, K.A.; Snurr, R.Q.; Hupp, J.T.; Farha, O.K. A facile synthesis of UiO-66, UiO-67 and their derivatives. *Chem. Commun.* **2013**, *49*, 9449.
2. Cavka, J.H.; Jakobsen, S.; Olsbye, U.; Guillou, N.; Lamberti, C.; Bordiga, S.; Lillerud, K.P. A new zirconium inorganic building brick forming metal organic frameworks with exceptional stability. *J. Am. Chem. Soc.* **2008**, *130*, 13850–13851.
3. Ren, X.; Li, R.; Song, Y.; Feng, X.; Wang, B. A mild-condition synthetic pathway to prepare POMs and MOF composites and performance of the composites for adsorb metal ions. *Sci. Sin. Chim.* **2014**, *44*, 1521.
